# Multi-flow channel bioreactor enables real-time monitoring of cellular dynamics in 3D engineered tissue

**DOI:** 10.1038/s42003-019-0400-z

**Published:** 2019-05-03

**Authors:** Barak Zohar, Yaron Blinder, Mark Epshtein, Ariel A. Szklanny, Ben Kaplan, Netanel Korin, David J. Mooney, Shulamit Levenberg

**Affiliations:** 1 Department of Biomedical Engineering, Technion-Israel Institute of Technology, Haifa, Israel; 2 Wyss Institute for Biologically Inspired Engineering at Harvard University, Boston, MA USA; 3 Bruce Rapaport Faculty of Medicine, Technion-Israel Institute of Technology, Haifa, Israel; 4 School of Engineering and Applied Sciences, Harvard University, Cambridge, MA USA

**Keywords:** Biotechnology, Angiogenesis, Tissue engineering, Biological models, Biophysical methods

## Abstract

The key to understanding, harnessing, and manipulating natural biological processes for the benefit of tissue engineering lies in providing a controllable dynamic environment for tissue development in vitro while being able to track cell activity in real time. This work presents a multi-channel bioreactor specifically designed to enable on-line imaging of fluorescently labeled cells embedded in replicated 3D engineered constructs subjected to different flow conditions. The images are acquired in 3D using a standard upright confocal microscope and further analyzed and quantified by computer vision. The platform is used to characterize and quantify the pace and directionality of angiogenic processes induced by flow. The presented apparatus bears considerable potential to advance scientific research, from basic research pursuing the effect of flow versus static conditions on 3D scaffolds and cell types, to clinically oriented modeling in drug screening and cytotoxicity assays.

## Abstract (web summary)

Zohar et al. demonstrate a real-time imaging of fluorescently labeled cells embedded in 3D constructs subjected to different flow conditions. This study provides a framework that enables tracking cell activity continuously during tissue development, suggesting its potential utility for drug screens or cytotoxicity assays.

## Introduction

Tissue engineering techniques typically make use of constructs, which generally involve 3D polymeric scaffolds in combination with one or more cells types, to form implantable tissue-like devices to replace damaged tissue. Additionally, engineered tissues can serve as models in the in vitro study of normal and diseased biological processes at the tissue level^1,2^. Vascularization of engineered tissue constructs in vitro is a challenge of great significance for regenerative medicine. Without a stable and perfusable blood vessel network to provide oxygen and nutrients, cells cannot survive once the tissue dimensions grow beyond several hundred microns due to diffusive limitations^3–5^. Thus, perfusion bioreactors play an important role in creating the specific culture conditions necessary for the development of 3D engineered tissues by improving nutrient transportation and waste removal and generate mechanical stimulation in the form of shear stress^6^. Direct perfusion culture has been shown to be beneficial for culturing various types of engineered tissues, such as cardiac^6–8^, hepatic^9,10^, cartilage, and bone tissue^11–13^, with even very low flow rates inducing widespread changes in gene and protein expression in multiple cell types^14,15^. Flow-induced shear stress has been shown to regulate and enhance angiogenic processes in microfluidic chips^16–19^ and monolayer two-dimensional (2D) flow-over models^20,21^. For example, direct flow-stimulated vascular morphogenesis and angiogenesis-related gene expression in 3D collagen and alginate gel plugs embedded with endothelial cells^22,23^. Recently, we reported that an interstitial-like, flow-induced, low and constant shear stress (0.75 dyne/cm^2^) enhanced vascular network formation and maturation in implantable 3D-engineered tissue^24^.

Although 3D culturing under dynamic conditions better mimics natural tissue environments, the vast majority of in vitro research is still conducted under static conditions, largely due to the ease of handling and low risk for contamination. In addition, microfluidic devices serving as perfusion platforms are still limited in geometry and scale, involve complicated fabrication techniques, and require designated facilities. Thus, direct flow bioreactors are currently the ultimate systems for cultivating clinically relevant engineered tissues, but are unsuitable for in situ imaging studies.

Hence, we developed the multi-flow view (MFV) bioreactor which enables real-time imaging of cellular dynamics in 3D constructs under flow conditions. The multichannel MFV bioreactor is designed to be loaded with up to four replicated 3D engineered constructs for long cultivation periods under diverse flow stimulations. The device is compatible with a standard upright confocal microscope for 4D scanning, which can be deployed to analyze cell dynamics.

In this study, the MFV is applied to monitor angiogenic processes in 3D-engineered tissue cultured under direct flow conditions. The platform allows for definition of the kinetics of microvascular formation in 3D-engineered tissue, as assessed by total vessel length, and the impact of flow on vascularization rate, measured by total vessel elongation and tip cell dynamics. In addition, the vascular network topology is characterized by tracking the structural angles during vascular network development. Furthermore, we demonstrate two additional models for exploring the dynamics of endothelial and supporting cells cultured under wall shear stress stimuli in a 3D fused construct and a tissue-engineered vascular graft (TEVG). In conclusion, the MFV bioreactor is a powerful platform that can be easily implemented to quantify and analyze cellular dynamics in 3D engineered tissues cultured under controlled flow conditions.

## Results

### Vasculogenic dynamics in a perfused 3D-engineered tissue construct

**Fig. 1:**
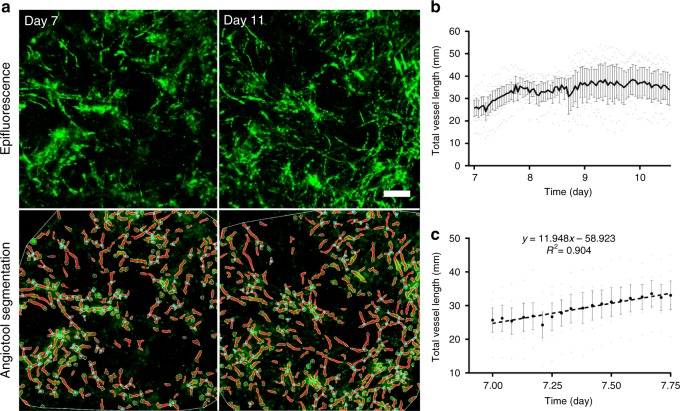
Vascular dynamics under direct flow conditions. Vascular network formation in constructs cultured for 5 days under static conditions, followed by 6 days of culture under direct flow conditions (0.1 mL/min) inside the MFV bioreactor. **a** HAMEC epifluorescence (green) and respective Angiotool segmentation (green = EC epifluorescence, red = segmented vessels, light blue = segmented junctions) in scaffolds 7 and 11 days post-seeding (scale bar—200 µm). **b** Trend of mean total vessel length formed in each scaffold as determined by Angiotool (*n* = 5 scaffolds). **c** Linear regression of the mean total vessel length during angiogenesis (day 7). Error bars represent standard error of the mean (SEM)

The formation of vascular networks within 3D-engineered tissue constructs has been described in a previous work, following real-time imaging of endothelial cells cultured under static conditions^25^. However, within the body, endothelial cells are also affected by biomechanical and biochemical stimulations induced by blood flow. By using the MFV bioreactor, we were able to visualize and quantify angiogenic processes as occur under flow-induced shear stress. Scaffolds were 3D scanned inside the MFV bioreactor every hour from day 7 to 11 post-seeding and the cells remained viable and sterile after 5 days of culture under flow conditions. Moreover, GFP-labeled HAMECs formed de-novo micro-vascular networks that were clearly visible for 3D image acquisition via confocal microscope (Fig. 1a and Supplementary Movie 1). A rapid increase in total vessel length was observed on day 7 post-seeding, followed by a stationary phase (Fig. 1b). On day 7, total vessel length rose approximately linearly (*R*^2^ ~0.9), at a constant elongation rate of ~12 mm/day (Fig. 1c), which then gradually declined to zero after day 9. The dynamic of the total vessel length can be explained by the biological mechanism of angiogenesis process. Angiogenesis refers to the expansion of existing vascular networks into new blood vessels via several mechanisms such as sprouting, intussusception (vessel splitting) and/or vessel fusion. Previous studies have shown that the process requires several types of specialized, distinctly differentiated endothelial cells. These include tip cells which lead the way using filopodia, stalk cells, which remain behind the tip cells and maintain the stalk of the vessel, and the quiescent phalanx cells, which line new vessel branches once they are integrated into the network^26,27^. Finally, tip cells anastomose with existing vessels and stop sprouting. Intuitively, the decreased total vessel elongation rate, during days 9–11, is affected by the increased probability of a tip cell to meet a pre-existing vessel, which increases as the total vessel length increases.

### The effect of direct flow-induced shear stress on angiogenesis dynamics

**Fig. 2:**
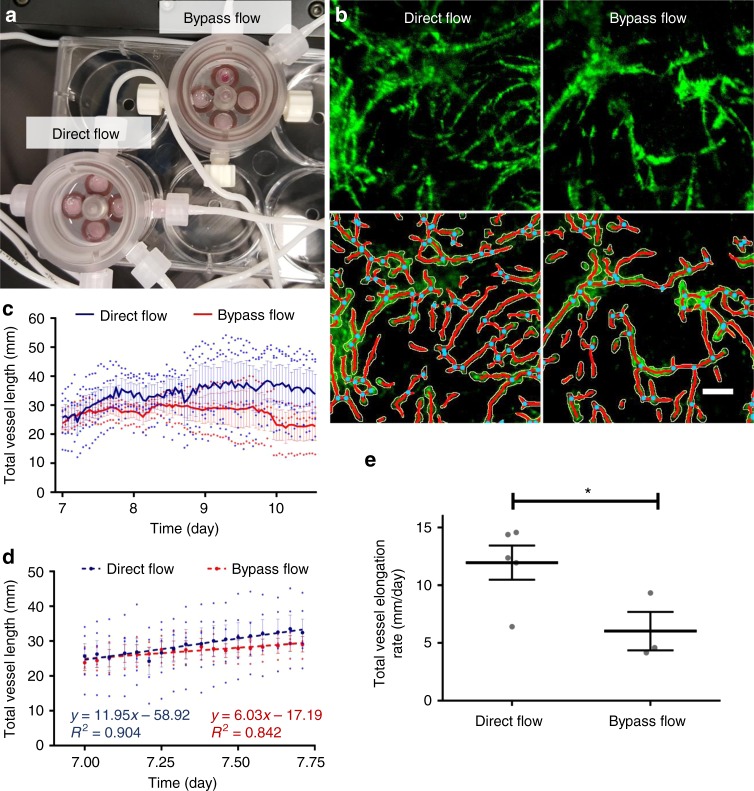
The effect of direct flow on vascular network formation kinetics. Vascular network formation in constructs cultured for 5 days under static conditions, followed by 6 days under direct flow (0.1 mL/min) or control conditions (bypass flow). **a** Picture of two MFV chamber set-ups for direct and bypass (control) flow conditions. **b** HAMEC epifluorescence (green) and respective Angiotool segmentation (green = EC epifluorescence, red = segmented vessels, light blue = segmented junctions) 7 days post-seeding, in scaffolds cultured under direct and bypass flow conditions (scale bar—200 µm). **c** Trend of mean total vessel length formed in each scaffold subjected to either direct or bypass (control) flow conditions, as determined using Angiotool. **d** Linear regression of the mean total vessel length in each scaffold subjected to either direct or bypass (control) flow conditions during angiogenesis (day 7). **e** Total vessel elongation rate was calculated as the mean slope obtained from replicated scaffold total vessel length linear regressions (*n* = 5 scaffolds for direct flow, *n* = 3 scaffolds for bypass flow). Error bars represent standard error of the mean (SEM). *Indicates statistical significance (*p*-value = 0.046)

The MFV bioreactor is a novel culture platform that can be applied for testing the effect of flow on cell activity in 3D culture. To demonstrate its capacities, we tested the effect of direct flow on the vascular elongation rate during angiogenesis occurring in 3D constructs. Control samples included pre-vascularized constructs subjected to the same culture conditions, but without direct flow-stimulated shear stress. In these samples (*n* = 3 scaffolds), medium was only circulated through a nearby bypass channel (Fig. 2a and Supplementary Movie 2). The mean of total vessel elongation rates measured as ~12 mm/day (*R*^2^ ~0.90) and ~6 mm/day (*R*^2^ ~0.84) for the flow and control conditions, respectively (Fig. 2d), amounting to a 100% increase (*p*-value = 0.046) in total vessel elongation rate upon shear stimulation (Fig. 2e). These findings corroborate with an earlier study, in which we demonstrated the significant impact of direct flow through 3D constructs on vessel formation, as manifested by a ~100% increase in total vessel length after 2 days of culture under flow conditions, *p-*value < 0.05^24^. These findings suggest that the enhanced vascularization is likely to be explained by a change in the kinetics of the angiogenic process.

**Fig. 3:**
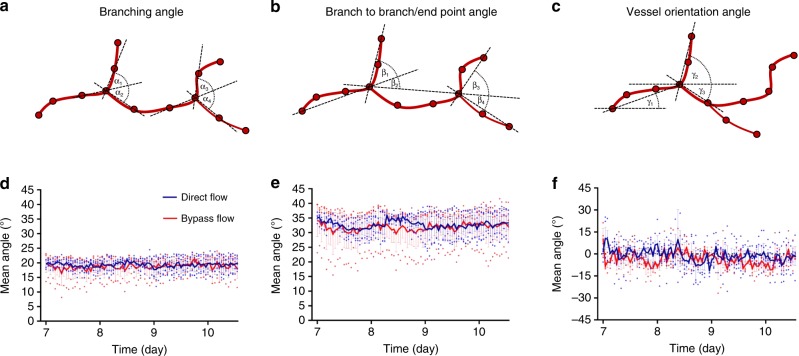
Vascular network topology analysis by IMARIS. **a** Drawing of vessel branching angle. **b** Drawing of vessel branch to branch/end point angle. **c** Drawing of vessel orientation angle. **d** Trend of mean vessel branching angle. **e** Trend of mean vessel branch to branch/end point angle. **f** Trend of mean vessel orientation angle (*n* = 4 scaffolds for direct flow, *n* = 3 scaffolds for bypass flow, 250–1000 angles were segmented for each scaffold). Error bars represent standard error of the mean (SEM)

Topological analysis of the newly formed vascular network (Fig. 3a–c) showed no significant change in mean angle measurements neither within 4 days of cultivation (from day 7 to day 11 post-seeding) nor under flow conditions, indicating that vascular network topology was maintained stable during angiogenesis (Fig. 3d–f). These findings stand in line with a study in yolk sacs of chicken embryos at two different developmental stages, reporting no measurable impact of hydrodynamic forces on branching angles during vascular network development^28^. Generally, in our study, the topology of the obtained vascular network showed an anisotropic structure. Although the theoretical mean angle for both branching and branch to branch/end point angles equally distributed between 0° and 180° was expected to be 90°, it reached ~20° and ~33° for branching and branch to branch/end point angles, respectively. Previously published mathematical analyses claim that this anisotropic structure is based on energy optimization principles influenced mainly by circumferential stresses that dictate the morphological construction of vascular trees^29,30^. Our findings align with the theoretical optimum of a symmetrical arterial bifurcation, as predicted by optimality principle of minimum surface^31^. Unlike the mean angle of both branching and branch to branch/end point angles, the mean vessel orientation angle indicated an isotropic structure. The mean vessel orientation angle reached ~0° as the expected theoretical mean angle equally distributed between −180° and 180°. These findings suggest a randomized vascular network structure that was both preserved during the angiogenesis process and unaffected by vertical direct flow conditions.

### Endothelial tip cell dynamics under flow conditions

**Fig. 4:**
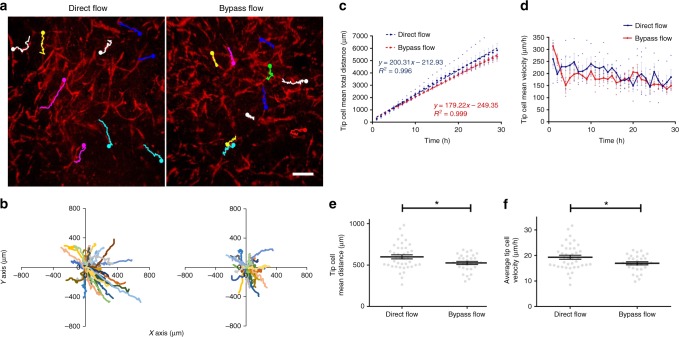
Endothelial tip cell tracking analysis. **a** EC epifluorescence (red) and respective manual tracking of endothelial tip cells (each tip cell path is presented by a different color) within the angiogenesis period (day 8) in scaffolds under direct flow and control conditions (scale bar—250 µm). **b** Multiple (*n* = 40 tip cells for direct flow, *n* = 30 tip cells for bypass flow, 10 tip cells for each scaffold) tip cell trajectories plotted from a shared origin point (each tip cell path is presented by a different color). **c** Trend and linear regression of mean overall tip cell distances and **d** trend of mean overall tip cell velocity within the angiogenesis period in each scaffold cultured under direct flow or control conditions, as measured with the NIH ImageJ manual tracking plug (*n* = 4 scaffolds for direct flow, *n* = 3 scaffolds for bypass flow). **e** Mean tip cell distance and **f** mean tip cell velocity during the angiogenesis period (*n* = 4 scaffolds for direct flow, *n* = 3 scaffolds for bypass flow). Error bars represent standard error of the mean (SEM). *Indicates statistical significance (*p-value = 0.019)

Pro-angiogenic signal such as a local gradient of VEGF induces angiogenesis by stimulating ECs to both sprout and branch from pre-existing vessels. Initially, ECs secrete matrix metalloproteases (MMPs) for degrading their basement membrane and starting a selection process known as tip cell selection. This NOTCH-DLL4-dependent selection process precedes to vascular sprouting^27,31^. Endothelial cells sprout within similar 3D constructs exhibited a tip-cell at their leading edge with visible filopodia that can be tracked manually^20^. To better understand how vascular network formation is accelerated by flow stimulation, we manually tracked tip cell positions in each individual sprout during angiogenesis, in constructs cultured under direct flow versus control conditions (Fig. 4a and Supplementary Movie 3). In a previous study applying the same manual tracking procedure, sprout directionality and speed distribution analyses in similar 3D construct cultured under static conditions indicated random endothelial sprouting directionality, with mean speed of 0.281 µm/min (16.86 µm/h)^20^. Similar observations were made here, where tip cell trajectories plotted from a shared origin point (Fig. 4b) indicated an isotropic sprouting structure under control (bypass flow) conditions. Furthermore, under these conditions, endothelial tip cells resulted in a sprouting mean speed of ~17 µm/h (Fig. 4f). In contrast, under direct flow stimulation, tip cell trajectories displayed extended and less isotropic sprouting structures (Fig. 4b). Both mean overall tip cell sprouting distance and tip cell mean velocity under flow conditions were increased as compared to those of the control tip cell (Fig. 4e, f). A mean sprouting speed of ~20 µm/h (Fig. 4f) was recorded in samples subjected to sheer stimuli, while the mean sprouting speed in control samples was ~17 µm/h. These findings indicate that flow-induced shear stress has a substantial impact on endothelial tip cell dynamics and consequently, on angiogenesis in the framework of neo-vascularization processes occurring in 3D-engineered tissue. Furthermore, the anisotropic sprouting structure may be an indicator of manipulated tip cell directionality, dictated by the flow stimuli.

### The effect of wall shear stress on endothelial cell rearrangement in a cross-sectional view

**Fig. 5:**
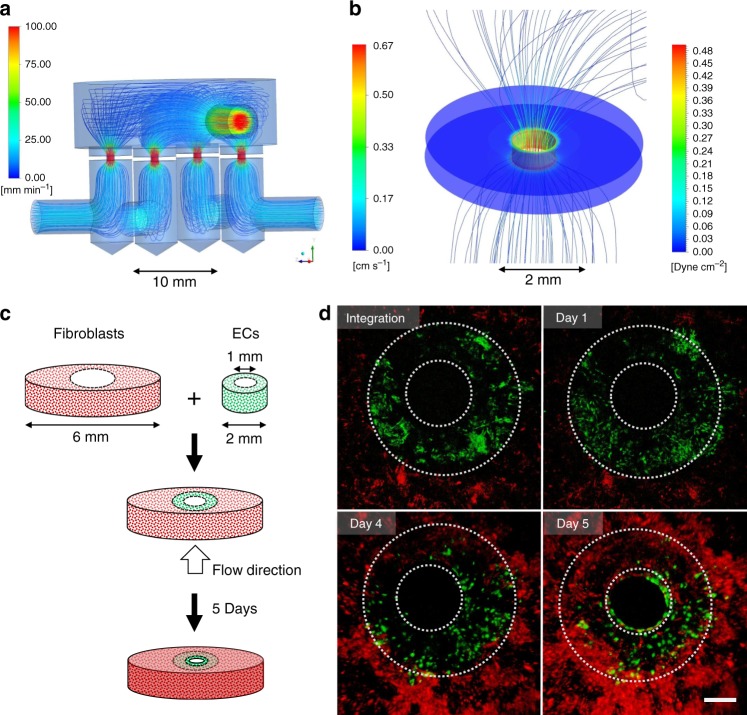
Flow-induced rearrangement of ECs in a fused macro-channel construct. **a** Flow pathlines in four fused macro-channel scaffolds placed inside the MFV chamber. The pathlines are color-coded by their velocity magnitude (mm/min—see scale bar), determined using FLUENT software. **b** Flow pathlines and wall shear stress inside a macro-channel (diameter of 1 mm) simulated by a volumetric flow rate of 0.1 mL/min (velocity (cm/s) and shear stress (dyne/cm^2^)—see scale bar), using the FLUENT software. **c** Scheme of the fabrication process of a fused macro-channel construct. **d** EC (green) and HNDF (red) epifluorescence in a fused macro-channel construct after constructs integration and after 1, 4, and 5 days of culture under flow conditions (scale bar—500 µm)

In attempt to monitor and characterize cell arrangement induced by wall shear stress, we generated a cross-sectional view of a macro-channel by creating a fused 3D construct with a 1 mm diameter central hole, composed of an inner part pre-seeded with ECs and outer part pre-seeded with HNDFs (Fig. 5c). We applied wall shear stress of ~0.3 dyne/cm^2^ to ensure both vascular network self-assembly and construct integrity during cultivation under flow conditions (Fig. 5a, b). To our knowledge, this is the first report of long-term cross-sectional viewing of vessel dynamics under flow. During cultivation inside the MFV bioreactor, HNDFs continued to proliferate massively and even penetrated and bridged the inner EC construct within 4 days of culture in the MFV bioreactor (Supplementary Figure 1A and 1C). Furthermore, after 5 days of culture, HNDFs were even observed creating a smooth and rounded new inner border along the inner frame of the construct (Supplementary Movie 4, Fig. 5c, Supplementary Figure 1B, C). ECs did not form the typical clusters, but rearranged in the new inner border created by the HNDFs and altered their morphology from rounded to a disc shape (Supplementary Movie 4, Fig. 5c, Supplementary Figure 1B).This EC behavior was observed only when flow was applied inside the macro-channel (Supplementary Movie 5, Supplementary Figure 2). Flow-induced shear stress has been shown to correlate with morphological changes in endothelial cells in vitro^32–35^. Consequently, the observed phenomenon may be indicative of a shear stress-induced cue for ECs to rearrange and form an endothelium structure.

### 4D imaging of EC arrangement into an endothelial structure in a flowing TEVG

**Fig. 6:**
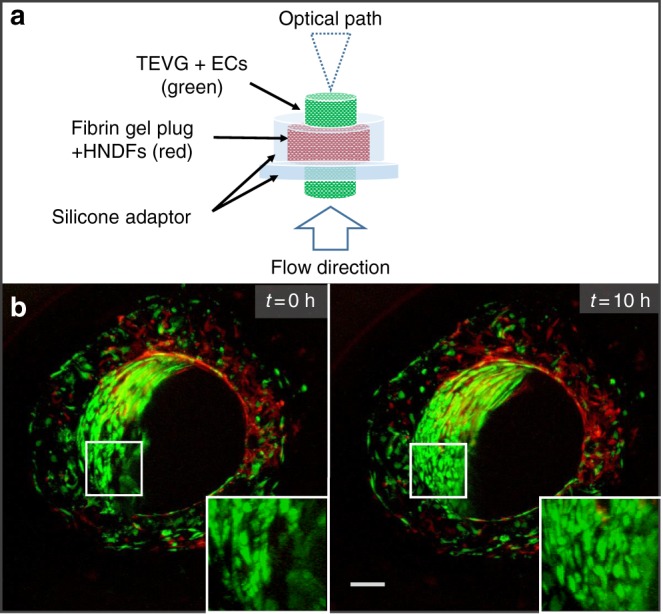
Flow-induced endothelial structure in a TEVG set-up. **a** Scheme of the tissue-engineered vascular graft (TEVG) set-up components. (scale bar—2.5 mm). **b** EC (green) and HNDF (red) epifluorescence in a flowing TEVG construct scanned in the MFV bioreactor before and after 10 h in culture under flow of 0.1 mL/min (scale bar—500 µm)

TEVG have a broad range of clinical applications. Although these grafts have demonstrated the ability to transform into functioning blood vessels, the underlying mechanisms remain to be elucidated^36^. Cultivation of a TEVG before transplantation requires dynamic culture conditions that mimic the physiological mechanical stimulations presenting in matured blood vessel. Therefore, the TEVGs are usually cultured in pulsatile flow bioreactors to both improve vessel mechanical properties^37–39^ and possess a confluent, adherent, and quiescent endothelium to resist thrombosis in vivo^40^. However, standard pulsatile flow bioreactors do not support live imaging of cells cultured inside the TEVG, specifically of ECs lining the lumen. In the following experimental set-up (Fig. 6a), we demonstrate a model of cultivating TEVGs under flow conditions inside the MFV bioreactor. 4D imaging of the early 10 h of cultivation inside the MFV bioreactor demonstrated for the first time, to our knowledge, the dynamics of ECs inside the lumen of a TEVG cultured under flow conditions (Fig. 6b and Supplementary Movie 6). The increased number of ECs lining the lumen of a flowing TEVG is indicated by the increased fluorescence area fraction measured in the lumen (Supplementary Figure 3).

### Conclusions

Perfusion bioreactors pass culture medium through engineered tissue constructs, thereby enhancing oxygen and nutrient availability throughout the entire construct volume, as well as providing for mechanical stimulation in the form of fluid shear stress. Yet, these culture apparatuses do not support real-time monitoring of cellular activity. Herein, we developed the MFV bioreactor, a multi-channel flow bioreactor which enables culture and visualization of fluorescently labeled cells in multi-3D-engineered tissues while applying flow stimulation. Here, the MFV was applied to explore the effect of flow-induced shear stress on ECs in different implantable 3D-engineered tissue models. Cultured in a 3D porous constructs under direct flow conditions, the ECs remained sterile and viable, and created micro- vascular networks that were remained stable throughout the 8 days of culture. During cultivation, images were acquired with no major optical interferences, and at resolution sufficient for visualization and analysis of EC motility and micro-vascular network formation. Flow-induced shear stress was shown to accelerate angiogenesis, as manifested by increased vessel elongation rate compared to control conditions. In parallel, vascular network topology remained stable during neo-vascularization and was unaffected by direct vertical flow stimulation. Interestingly, quantification of tip cell dynamics revealed that the accelerated angiogenesis can be partially explained by increased tip cell velocity. We further utilized the device to explore EC rearrangement and proliferation in response to flow in macro-channel and TEVG models. In conclusion, the MFV bioreactor enables real-time monitoring of cellular dynamics in response to controlled and versatile biochemical and biomechanical stimulations in replicated versatile 3D-engineered constructs. Consequently, we believe that the MFV will be a valuable tool in addressing a wide range of biomedical questions.

## Methods

### Cell culture

Zs-green-expressing human adipose microvascular endothelial cells (HAMECs) (Passage 5–7, ScienceCell) were cultured in tissue culture flasks. HAMECs were cultivated in endothelial cell medium (ScienceCell), supplemented with 5% fetal bovine serums (FBS) (ScienceCell) and endothelial cell growth supplement (ScienceCell). Neonatal human dermal fibroblasts (HNDFs) (Passage 6–8, Lonza) were cultured on tissue culture flasks in DMEM (Gibco) supplemented with 10% fetal bovine serum (FBS) (Hyclone), 1% non-essential amino acids, 0.2% β-mercaptoethanol, and 1% penstrep (Sigma Aldrich).

### Vascular 3D porous construct preparation

Macroporous poly-l-lactic acid (PLLA) (Polysciences, Warrington) and poly-l-glycolic acid (PLGA) (Boehringer Ingelhein) (weight ratio of 1:1) scaffolds were prepared using a porogen leaching protocol as previously described^41^ and finally cut to a final disc-shaped construct (diameter of 6 mm and thickness of 1 mm). ECs and HNDFs were mixed and seeded upon the scaffolds (500,000 and 100,000 cell, respectively, per scaffold) in 14 μL human fibrin gel, prepared from a mixture (volume ratio of 1:1) of thrombin solution (15 mg/mL, Johnson & Johnson Medical, Israel) and human fibrinogen solution (5 mU/mL, Johnson & Johnson Medical). Co-culture medium based on both two-cell media (a 1:1 mixture) was added (2 mL per scaffold) after 30 min of incubation (37 °C, 5% CO_2_). Scaffolds were then cultured under static conditions on a 12-well non-tissue culture plate (37 °C, 5% CO_2_) for 5 days before being loaded in the MFV bioreactor. Co-culture medium was replaced every 2–3 days.

### Fused macrochannel construct preparation

HNDFs (100,000 cell per scaffold) and HAMECs (500,000 cell per scaffold) were seeded separately in 3D monoculture constructs, as was described above. After 2 days of culture under static conditions, constructs embedded with HAMECs were cut into ring shapes (outer diameter of 2 mm and inner diameter of 1 mm, cut using a biopsy punch) and manually fused, using sterile forceps, into constructs embedded with HNDFs that were cultured for 5 days under static conditions as illustrated in Fig. 5c.

### TEVG preparation

Tubular porous scaffolds (inner diameter of 2.5 mm) were prepared as previously described, with several modifications^42^. Briefly, a 5% (wt/vol) solution of PLLA (Polysciences Inc.) in chloroform was prepared. NaCl particles were ground and sifted with a 125 µm pore sieve. NaCl particles (1 mL) were then mixed into 5 mL of the PLLA solution. A 2.5-mm-diameter stainless steel rod was dipped into the NaCl:PLLA mixture for 10 s and then air-dried at room temperature for 2 min. This formed a NaCl:PLLA layer surrounding the metal rod. The rod was then immersed in methanol for 30 s to facilitate the separation of the overlaying NaCl:PLLA layer from the rod. The tubular NaCl:PLLA layer was removed and placed overnight in distilled water, to remove the NaCl particles. Scaffolds were then dried at room temperature for 2 h and cut to a length of 10 mm. Porous scaffolds were disinfected by immersing them in ethanol for 30 min. The inner surface of the tubular scaffolds was coated using a 50 µg/mL human fibronectin solution (Sigma) for 1 h at 37 °C. After rinsing the remaining unbound fibronectin with PBS, 15 µl HAMEC suspension with 5 × 10^6^ cell/mL were seeded into the lumen of the scaffold. To achieve a uniform cell lining, the scaffolds were incubated at 37 °C for 2 h under axial rotation. The non-attached cells were washed out with HAMECs medium. Tubular scaffolds were cultured under static conditions (37 °C, 5% CO_2_) for 2 days and then placed through a perforated silicon cup-like holder filled with 5 × 10^4^ HNDFs suspended in fibrin (Fig. 6a). Constructs were cultured in co-culture medium for one additional day under static conditions before been loaded in the MFV bioreactor.

### MFV bioreactor design

**Fig. 7:**
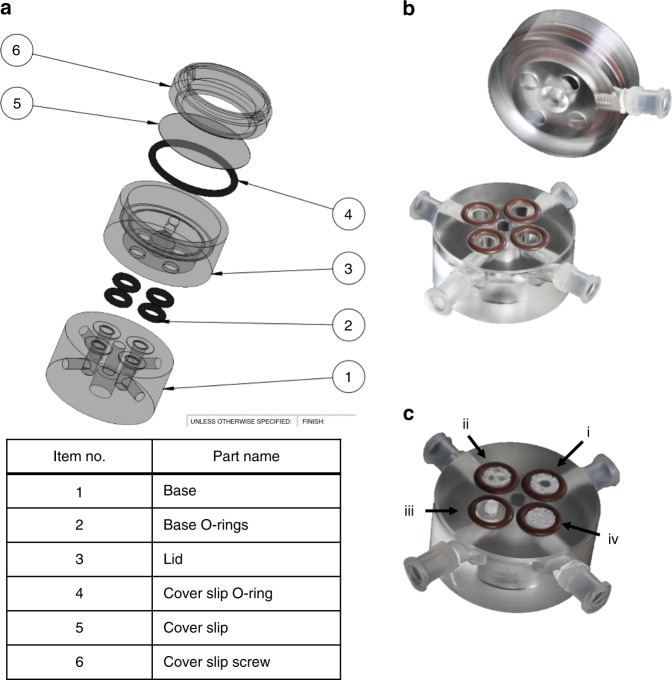
Multi-flow view (MFV) chamber. **a** Drawing of the MFV chamber parts (SolidWorks^®^). **b** Picture of the assembled MFV chamber base and lid parts. **c** Picture of the MFV chamber loaded with single-holed (i), multi-holed (three holes, ii), vessel like (with PDMS holding ring, iii), and standard scaffolds (iv)

**Fig. 8:**
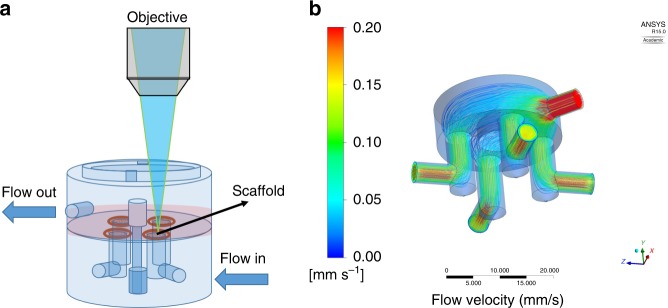
MFV bioreactor setup. **a** Schematic diagram of single MFV bioreactor perfusion culture system. **b** Picture of two MFV bioreactor perfusion culture systems located inside a temperature-controlled chamber of an upright confocal microscope

The MFV chamber (35 mm × 35 mm) is uniquely designed to enable in-situ imaging using a standard upright microscope. It is comprised of base and lid parts made of poly(methyl methacrylate) (PMMA) or poly(ether ketone) (PEEK) (autoclavable version), a round 30 mm glass cover slip (0.15 mm thickness, Thermo Scientific), and Viton^®^ O-rings. The apparatus is designed to be placed in a standard six-well plate and loaded with four scaffolds placed in separated flow channels (Fig. 7a, b). Each MFV chamber is connected to a closed loop perfusion system (Fig. 8a), designed to be placed inside standard upright confocal microscope chamber (Fig. 8b). The tubing used to connect the system is made of a thermoplastic elastomer (ID0.031 X OD0.094, C-FLEX^®^, Cole-Parmer) and the tubing for the istaltic pump is made of PharMed^®^ BPT 1.14 mm ID RED/RED, ISMATEC)). These tubings were chosen because they are both autoclavable and have low CO_2_ and O_2_ permeability. The bioreactor is placed inside the temperature-controlled chamber (37 °C) of the microscope. To control physiological pH levels (7.2–7.4) inside the MFV chamber and avoid medium evaporation, air with 8% CO_2_ is bubbled by a sintered sparger through warm ultra-pure water. The gas mixture is filtered and supplied directly to the medium reservoir in overlay gassing mode (Fig. 8a).

### MFV bioreactor sterilization and handling

The MFV bioreactors were washed with purified water and PBS followed by sterilization in an autoclave (121 °C for 30 min). When using the PMMA chamber, it was disinfected by soaking in 70% volume fraction ethanol for 0.5 h and rinsed twice with PBS. The chamber was further assembled and aseptically connected to the sterile perfusion system in a laminar flow hood. To test the system integrity, the MFV bioreactors were incubated with the relevant culture medium, which was circulated for at least 24 h before scaffold loading. 3D scaffolds were placed aseptically (in a laminar flow hood) inside the MFV chamber and 20 mL (5 mL per each scaffold) of relevant co-culture medium was added to the reservoir bottle. Then, the MFV bioreactors were placed inside the confocal chamber, the reservoir bottles were connected to a gas supply and the flow chambers were fixed to the confocal robotic plate (Fig. 8b). During the cultivation period, the medium in the reservoir bottle was replaced every 72 h.

### Validation and characterization of culture conditions

**Fig. 9:**
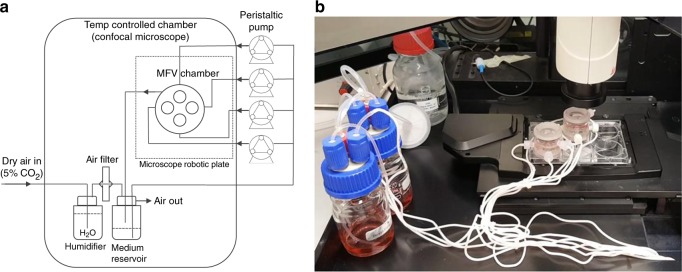
Flow characterization. **a** Drawing of a MFV chamber with flow and optics orientation. **b** Flow pathlines inside the MFV chamber, colored using the velocity magnitude scale (mm/s), as determined by FLUENT software

During the cultivation period, culture medium temperature and pH were checked daily and maintained within the physiological range of 36–37.5 °C (Brannan mercury thermometer) and pH 7.2–7.4 (Mettler Toledo pH meter). In addition, culture medium volume was measured after 3 days to ensure negligible decrease in working volume caused by water evaporation. Flow velocity inside the MFV chamber was characterized by a computational fluid dynamics (CFD) model to ensure uniform flow distribution within the separated channels (Fig. 8b). Steady-state CFD simulations were performed in FLUENT^®^ using the pressure-based coupled solver. The selected mesh was constructed in GAMBIT on the basis of the MFV chamber CAD model (Fig. 7a) and contained 1 × 10^6^ cells. The maximum Reynolds number estimated in the system was 4.5, so laminar flow was assumed. The maximum Damkohler number estimated in the system was 1.26 × 10^−5^ for oxygen consumption vs. transportation rate. The very low Damkohler number indicated that there is no nutrient transportation limitation under the applied flow conditions. The results were post-processed in the native Ansys^®^ post-processor. During cultivation time, a flow rate of 0.1 mL/min, corresponding to a mean shear stress of 0.75 dyne/cm^2^, was set for each flow channel separately (Figs. 8a and 9a)^24^. The same flow rate (0.1 mL/min) was also set for the fused macrochannel construct. Flow velocity and wall shear stress were characterized by a CFD model using the FLUENT software (Fig. 5b).

### Image acquisition and processing

For each scaffold, the initial position of the robotic plate was set to the center of mass of the scaffold for acquiring 2.5 × 2.5 mm (width of view) images using the Leica PLANAPO ×2.0/WD 39 mm objective. The z-stack was only defined after any medium replacement, to capture the maximum depth of field (400–600 µm, split into at least 30 z-stacks). The scaffolds were scanned in time lapse over 1 h, by a LEICA SuperZoom Z6 confocal upright microscope. 4-D (XYZT) confocal z-stacks were converted to 2D time-series TIFF stacks by performing z-projections at each time step using the NIH ImageJ software. Then, images were processed by contrast enhancement (stack histogram equalization and normalization, 0.4% saturated pixels).

### Image analysis

Each scaffold and frame were identically processed using the Angiotool^®^ interface^43^ and by applying the IMARIS fully automated FilamenTracer software (IMARIS 8.2.0), to quantify total vessel length and branching angles, respectively. Total vessel elongation rate was defined as the slope of the total vessel length trend, calculated by performing linear regression. Vascular branching angle, calculated by IMARIS, was defined as the angle between the extending lines connecting the branch point with neighboring points (Fig. 3a). Vascular branch to branch/end point angles were defined as the angles between the extending lines connecting the branch point with the neighboring branch points and the terminal points (Fig. 3b). Vascular orientation angle was defined as the angle formed between extending line connecting distal vertices of the vessel segment and *X*-axis of image within *XY* plane (Fig. 3c). When measuring the branching angles, only the close vicinity of the junction was considered and the origin of the parent artery and the destination of its branches were ignored. The mean angles were calculated as the mean of the average of 250–1000 angles segmented in each scaffold. Tip cell tracking was performed using the NIH ImageJ software manual tracking plug. The tip cell was identified as the end point of a vessel structure which continuously elongated over time. Tracking was done blindly for 10 randomly selected tip cells in each scaffold. Fluorescence area fraction was measured using the NIH ImageJ software.

### Statistics

Measurements were performed in triplicates, at minimum, and images were scanned, processed, and analyzed using an identical setup. Means were plotted, with error bars representing the standard error of the mean (SEM). Statistical comparisons were performed using the Student’s *t*-test with a 95% confidence limit (two-tailed and unequal variance). Differences with a *p*-value < 0.05 were considered statistically significant.

### Reporting summary

Further information on experimental design is available in the Nature Research Reporting Summary linked to this article.

## Supplementary information

Reporting Summary

Supplementary Information

Supplementary Data 1

Description of Additional Supplementary Files

Supplementary Movie 1

Supplementary Movie 2

Supplementary Movie 3

Supplementary Movie 4

Supplementary Movie 5

Supplementary Movie 6

## Data Availability

The data that support the findings of this study are available from the corresponding author upon reasonable request. The source data underlying the graphs and charts presented in all figures are in the Supplementary Data 1.
