# Supplementary material for: Multi-flow channel bioreactor enables real-time monitoring of cellular dynamics in 3D engineered tissue
Source: Commun Biol. 2019 May 3;2:158. doi: 10.1038/s42003-019-0400-z (PMC6499812; doi:10.1038/s42003-019-0400-z)
Supplement: Supplementary file 2 — Supplementary Information [file 42003_2019_400_MOESM2_ESM.pdf]

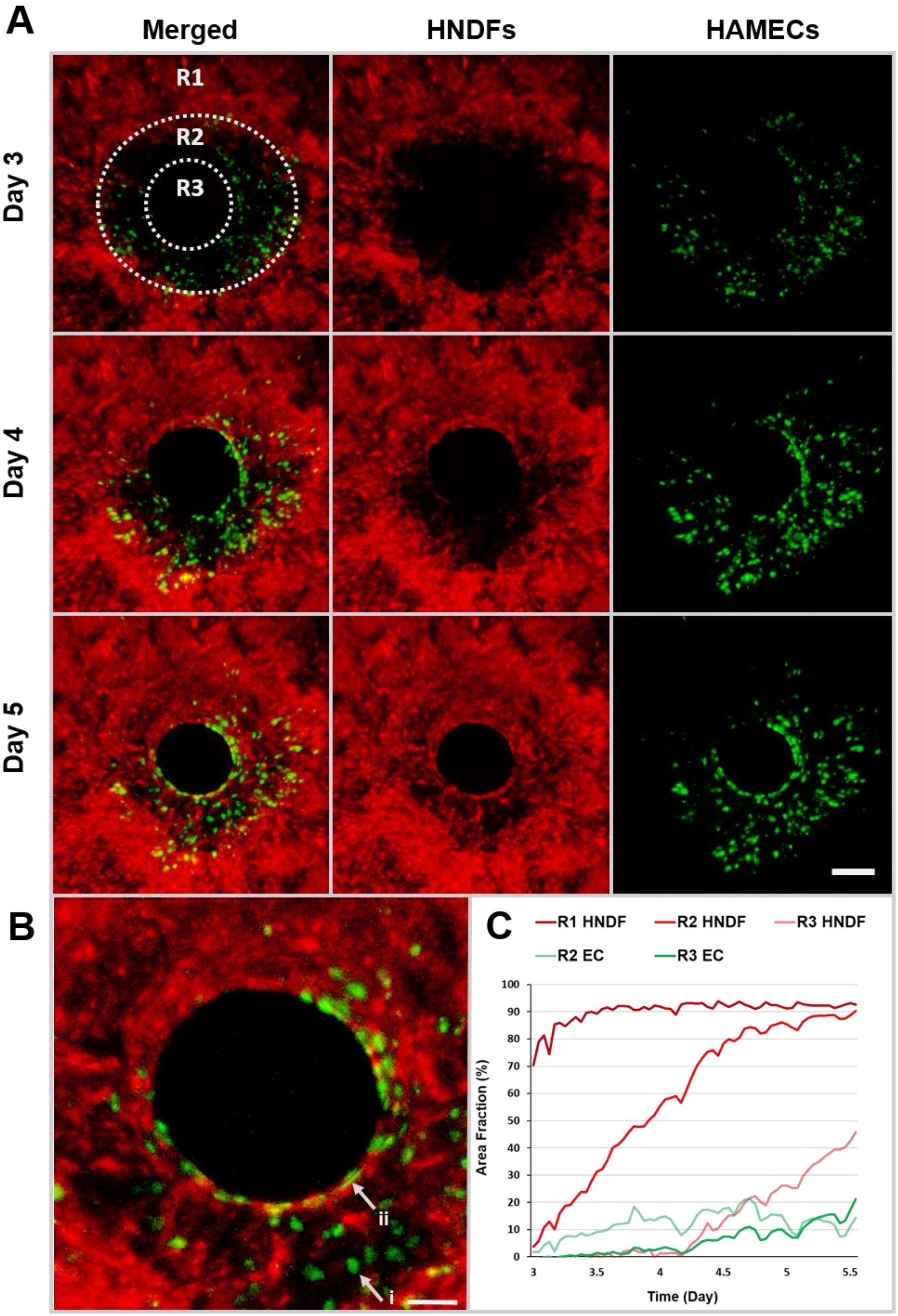

A
Merged
HNDFs
HAMECs
Day 3
Day 4
Day 5
R1
R2
R3
B
ii
i
C
R1 HNDF
R2 HNDF
R3 HNDF
R2 EC
R3 EC
Area Fraction (%)
Time (Day)

Supplementary Figure 1. (A) HAMEC (green) and HNDF (red) epifluorescence in a fused macro-channel construct after 3, 4 and 5 days of culture under flow conditions (scale bar - 500μm). In the upper left image, R1 refer to the outer scaffold area, R2 to the inner scaffold area and R3 to the inner hole. (B) Zoom into a macro-channel after 5 days of culture under flow conditions (scale bar - 200μm). Arrows are pointing on rounded (i) and disc shape (ii) EC morphology. (C) HAMEC (green) and HNDF (red) epifluorescence area fraction in R1, R2 and R3 areas within cultivation time under flow condition.

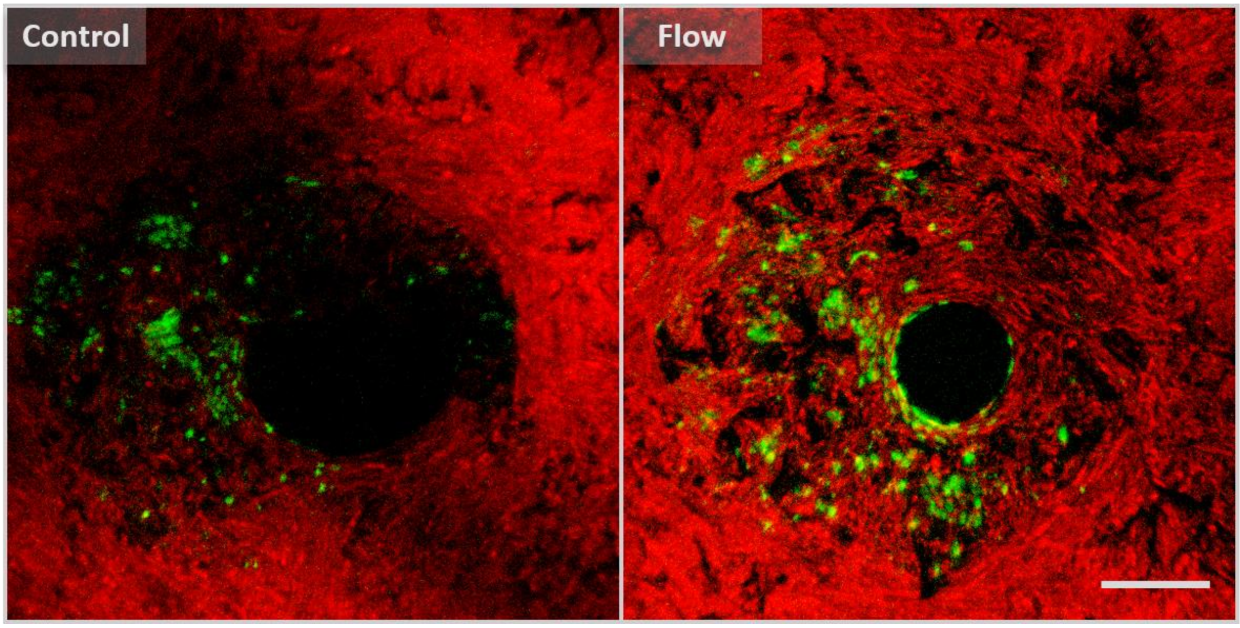

Supplementary Figure 2. HAMEC (green) and HNDF (red) epifluorescence in fused macro-channel constructs after 7 days of cultured under flow (0.1ml/min) and bypassed (control) conditions (scale bar - 500μm).

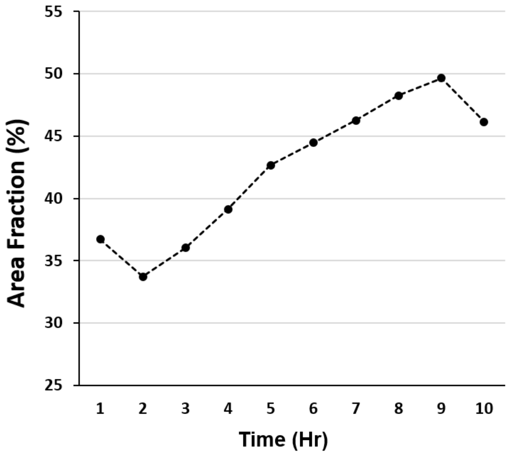

Supplementary Figure 3. HAMEC (green) epifluorescence area fraction measured in the lumen of a TEVG construct cultured under flow of 0.1ml/min.
