# Supplementary material for: Multi-flow channel bioreactor enables real-time monitoring of cellular dynamics in 3D engineered tissue
Source: Commun Biol. 2019 May 3;2:158. doi: 10.1038/s42003-019-0400-z (PMC6499812; doi:10.1038/s42003-019-0400-z)
Supplement: Supplementary file 4 — Description of Additional Supplementary Files [file 42003_2019_400_MOESM4_ESM.pdf]

Description of Additional Supplementary Files

File Name: Supplementary Data 1

Description: Source data underlying the graphs and charts presented in all figures

File Name: Supplementary Movie 1

Description: ECs epifluorescence (green) and Angiotool segmentation (green - 3 ECs, red-vessel segmentation and blue –junction segmentation) of 4 replicated 3D constructs 4 (Channel 1-4) scanned every hour within 4 days of cultivation under flow conditions in the MFV 5 bioreactor (scale bar - 200µm ,10 frames per second).

File Name: Supplementary Movie 2

Description: ECs epifluorescence (red) of 3D constructs scanned every hour within 7 4 days of cultivation in two separated MFV bioreactors: direct flow (Channel 1-4) and bypass 8 flow (control, channel 5-7) conditions (scale bar - 250µm, 5 frames per second).

File Name: Supplementary Movie 3

Description: EC epifluorescence (red) and respective endothelial tip cell manual 10 tracking (each tip cell is presented by a different color) within angiogenesis period (day 8) in 11 scaffolds under direct flow and control conditions (scale bar - 250µm, 5 frames per second)

File Name: Supplementary Movie 4

Description: EC (green) and HNDF (red) epifluorescences in a fused macro 13 channel construct tracked for 4 days of culture under intra-channel flow conditions (scale bar - 14 250µm, 5 frames per second)

File Name: Supplementary Movie 5

Description: EC (green) and HNDF (red) epifluorescence in fused macrochannel 16 constructs tracked for 6 days of culture under bypass (control) and flow (0.1ml/min) conditions 17 (scale bar - 500µm, 15 frames per second)

File Name: Supplementary Movie 6

Description: EC (green) and HNDF (red) epifluorescences in a TEVG set up 19 within 10 hours of cultivation under flow conditions (scale bar - 500µm, 5 frames per second)
